# Psychological Aspects Related to Diabetes Mellitus

**DOI:** 10.1155/2016/7276403

**Published:** 2015-12-07

**Authors:** Nitin Gupta, Sanjay Kumar Bhadada, Viral N. Shah, S. K. Mattoo

**Affiliations:** ^1^Department of Psychiatry, Government Medical College and Hospital-32, Chandigarh 160030, India; ^2^Department of Endocrinology, Postgraduate Institute of Medical Education and Research, Chandigarh 160012, India; ^3^Barbara Davis Center for Diabetes, School of Medicine, University of Colorado Denver, Aurora, CO 80045, USA; ^4^Department of Psychiatry, Postgraduate Institute of Medical Education and Research, Chandigarh 160012, India

The prevalence of diabetes has shown an exponential worldwide rise in recent years [1]. Poor glycemic control results in long-term micro/macrovascular complications, and thus most diabetes organizations recommended good glycemic control (defined as A1c less than 7%) to prevent these complications. However, management of diabetes requires lifelong daily adherence to dietary and exercise plans, frequent blood glucose monitoring, and adherence to medications. This results in higher risk for reduced physical, emotional, and social well-being (in terms of quality of life) among people with diabetes.

Over the decades, there has been a burgeoning research interest in the psychological aspects related to diabetes. Numerous evidences suggest the important role of psychosocial factors in diabetes self-management. Psychosocial problems can result in nonadherence to medications, poor quality of life, and lack of interest in managing disease resulting in poor glycemic control and long-term complications. In this regard, therefore, the American Diabetes Association (ADA) and various other diabetes organizations recommend psychosocial assessment of people with diabetes to improve diabetes related health outcomes [2]. In keeping with the quite broad and complex nature of diabetes, the research issues are numerous and varied. This special issue is in no way different. When the publishers and the editorial team had jointly envisaged this theme (and special issue) a year back, we had not anticipated the interest it would generate. An overwhelming number of submissions were received for this issue within a short span of just six months, after undergoing an extremely rigorous process of in-house and external peer review.

Various aspects related to diabetes have been reported in this special issue of the journal, namely, study of preventive factors, etiology, quality of life (QOL), clinical and psychosocial correlates, comorbidity, adherence issues, management (both self-intervention and external intervention based), and cultural issues.

The aforementioned psychological factors can influence the self-management of diabetes. In this issue, E. J. Dill et al. studied the effect of psychosocial factors such as psychological distress, coping skills, and family support on a weight loss program for the prevention of diabetes among 3135 American Indians and Alaska Natives. They demonstrated that psychosocial factors influence weight loss. It may be possible that addressing psychosocial factors might increase the success of diabetes prevention strategies.

Studies by M. M. A. Eilander et al. and M. P. Günther et al. demonstrated that behavior problems and psychosomatic factors are associated with glycemic control (reflected by A1c) in children and adolescent with type 1 diabetes (T1D). M. S. D'Souza et al. have studied determinants of the quality of life (QOL) among 300 adults with type 2 diabetes (T2D) from Oman. They showed that QOL is linked with self-management strategies; additionally management and knowledge of diabetes were higher in females. L. C. Jones et al. showed that 20% of 246 community-dwelling older adults (≥65 years) with T2D had depressive symptoms, with positive association between higher level of diabetes distress and depression. They conclude that if interventions are targeted at reducing the diabetes related distress and additional health complications arising out of diabetes, then one may be able to reduce depressive symptoms in patients with diabetes.

In a study by L. Wisting et al., they showed that eating disorder psychopathology and illness perception were important contributors to metabolic (glycemic) control in females with T1D. Studies from three different continents support the recommendations of the ADA that evaluation of psychosocial factors should be a part of all diabetes clinic visits [2], in order to improve diabetes related outcomes and QOL in these patients.

Significant research is being undertaken to understand the etiology of diabetes and diabetes prevention. The success of longitudinal epidemiologic studies lies with participant retention in such studies. B. Lernmark et al. have analyzed the factors associated with participant dropout in a large multinational TEDDY (The Environmental Determinants of Diabetes in the Young) study aimed at characterizing environmental factors causing T1D in children. Demanding research protocol, frequent blood draws, overwhelming research information, and time constraints were common factors related to participant dropout. The results so obtained made them advice caution regarding use of painful procedures, time required for participation, and assessment of study satisfaction. Thus, one can see that participation (and conversely withdrawal) from trials by patients with diabetes potential (and their families) can be influenced by various psychosocial factors.

Keeping in view the inherent nature of the disease and its long-term management, coupled with the individualistic and autonomous pattern of living in the West [3], which is now being increasingly seen in the Eastern/traditional countries [3, 4], self-management of diabetes assumes importance of significant proportions. In this regard, studies are available in this issue, which have examined this concept using differing research methodologies.

A.-R. Abubakari et al. studied role of various factors to explain adherence to self-management recommendations among over hundred people with poorly controlled diabetes by administering various questionnaires. They determined that the “illness perceptions” and “self-efficacy beliefs” of such patients were important predictors of their self-management behaviors and could potentially guide effective interventions.

M. Hofmann et al. specifically focus on adults with T2D and attempt to measure the impact of an internet-based, self-management intervention (“HeLP-Diabetes”) by mixed-methodology. The qualitative and quantitative data so generated demonstrated that the interventions positively impact both psychological and behavioral outcomes in these patients. However, it is necessary to bear in mind that the sample comprised only 19 participants.

The write-up by A. Jones et al. is, strictly speaking, not a research study but a “practical guide” for diabetes healthcare providers on the processes and techniques required for establishing a “working alliance” with patients having diabetes in order to enhance their self-management and positively influence their treatment outcome in relation to psychological and somatic aspects of the illness.

Recently, there has been focus in creating semiautomatic insulin delivery system (artificial pancreas, AP) to improve glycemic control and prevent long-term diabetes related complications, especially in patients with T1D [5]. However, the success of the AP lies in not only creating devices but also understanding factors associated with its acceptance. Apart from self-management, psychological aspects tend to be associated with any form of intervention for diabetes [6]. Hence, it is pertinent that this issue carries studies highlighting this key aspect too. C. Ziegler et al. studied the parameters of fear, satisfaction, and acceptance of AP system among patients with T1D. They demonstrated that the AP system was associated with reduced hypoglycemia worries and increased satisfaction in patients with T1D. However, this study was limited by small number of patients with T1D and was conducted over a very short duration of only 4 days.

With increasing advances in technology, patients with diabetes tend to access information regarding the illness and interventions online more frequently [7]. In another study from Netherlands, Y. Roelofsen et al. have investigated clinical and psychological characteristics between users and nonusers of an online platform. Over 600 patients with T2D were evaluated, and it was seen that patients who accessed the online platform had more favourable psychological characteristics (higher quality of life, better well-being, lesser distress, and better medication adherence). Hence, patients with poorer psychological profile tended to be more “unreached.” This study does have significant implications for not only planning interventions, but also reaching out to these patients.

Lastly, the impact of culture cannot be emphasized enough; it tends to influence personalities, behaviors, illnesses, and so forth [8]. Hence, the article by N. R. Patel et al. on the migrant British South Asians is quite topical and pertinent. Not only do they focus on this ethnic group (which has a disproportionately high prevalence of diabetes) in a Western country (i.e., UK), but also another cultural variable is studied in detail that is “impact of travel back to the East.” There is a qualitative study on 44 participants with both types of DM being interviewed cross-sectionally. They concluded that despite living in the UK, social networks in the East were very important for both information and support.

The World Health Organization has pledged to build awareness towards the global epidemic of diabetes [9]. To this end, assimilating information on the psychological aspects of diabetes in a comprehensive and scientifically critical manner shall be a step in right direction. Mainstream focus and interest in research and clinical aspects of diabetes have invariably centered around the physical aspects/complications. It is probably an opportune moment to provide the same focus and intensity to the psychological aspects too. Hence, understanding the pertinent psychological aspects related to diabetes is essential [6].

In a recent review in the World Journal of Diabetes, Chew et al. [6] have highlighted the need for more research to understand various individual (read as “psychological”) factors, cross-disciplinary working, and international collaboration. As editors of this special issue, we could not agree more with this. Additionally ADA, in the position statement, has highlighted the key role of team approach and collaborative care interventions [2].

We hope that this “special issue” shall stimulate the readers into not only furthering research on diabetes and related psychosocial aspects, but also developing service delivery models and higher standards of clinical care using a multidisciplinary team based integrated liaison model approach.

*Nitin Gupta* *Nitin Gupta* *Sanjay Kumar Bhadada* *Sanjay Kumar Bhadada* *Viral N. Shah* *Viral N. Shah* *S. K. Mattoo* *S. K. Mattoo*
